# Stromal Vascular Fraction in Osteoarthritis of the Knee

**DOI:** 10.3390/biomedicines11051460

**Published:** 2023-05-16

**Authors:** Madhan Jeyaraman, Nicola Maffulli, Ashim Gupta

**Affiliations:** 1 Department of Orthopaedics, ACS Medical College and Hospital, Dr. MGR Educational and Research Institute, Chennai 600056, Tamil Nadu, India; madhanjeyaraman@gmail.com; 2 Department of Biotechnology, School of Engineering and Technology, Sharda University, Greater Noida 201310, Uttar Pradesh, India; 3 South Texas Orthopaedic Research Institute (STORI Inc.), Laredo, TX 78045, USA; 4 Department of Musculoskeletal Disorders, School of Medicine and Surgery, University of Salerno, 84084 Fisciano, Italy; n.maffulli@qmul.ac.uk; 5 San Giovanni di Dio e Ruggi D’Aragona Hospital “Clinica Orthopedica” Department, Hospital of Salerno, 84124 Salerno, Italy; 6 Barts and the London School of Medicine and Dentistry, Centre for Sports and Exercise Medicine, Queen Mary University of London, London E1 4DG, UK; 7 School of Pharmacy and Bioengineering, Keele University School of Medicine, Stoke on Trent ST5 5BG, UK; 8 BioIntegrate, Lawrenceville, GA 30043, USA; 9 Future Biologics, Lawrenceville, GA 30043, USA; 10 Regenerative Orthopaedics, Noida 201301, Uttar Pradesh, India

In the United States, osteoarthritis (OA) affects 30 million people among the population and poses a major disability and financial burden that impact functional quality of life among the affected individuals [1]. OA knee, the joint that most commonly presents OA, contributes 80% of global cases. Many consensus statements on the management of OA knee are available, dwelling on a conservative approach to surgical management. Intra-articular injections are popular among orthopedic surgeons, as they are perceived to have a favorable risk–benefit ratio. These intra-articular injections (steroids, hyaluronic acid, and prolotherapy) can provide immediate and short-term pain relief with an improved range of movement across the knee joint [2,3]. With the revolution of Industry 5.0 (personalization and customization) [4], Tissue Engineering and Regenerative Medicine (TERM) are intended to provide a more suitable platform with which to explore results and functional outcomes following the administration of mesenchymal stem/stromal cells (MSCs) and their by-products [5]. Clinicians and researchers use various sources of MSCs according to their nations’ clinical regulations to manage patients with knee OA. In this annotation, we trace the evidence-based analysis of stromal vascular fraction (SVF) in the OA knee. 

An understanding of adipose tissue biology requires expertise. Adipose tissue has traditionally been considered as medical waste when performing liposuction in bariatric procedures, but researchers have worked on its regenerative potential in animal models, demonstrating its safety and efficacy in various diseases. Sharma et al. described the by-products of adipose tissue with regenerative potential, including adipose-tissue-derived MSCs (AD-MSCs), microfat, nanofat, SVF, microvascular fragments (MVF), and AD-MSC-derived exosomes and secretomes [6]. Given the presence of a heterogeneous population of cells in SVF, there is no need for the culture expansion of cells, which further decreases the risk of culture-induced chromosomal aberrations. Orthopedic surgeons and researchers need to undergo adequate training in order to understand and exploit the complex biology, isolation, and characterization of SVF before using it for the management of musculoskeletal conditions. The available literature on autologous adipose-tissue-derived SVF for the OA knee demonstrates the existence of level 1 evidence obtained through various network meta-analyses and systematic reviews, but controversies are still widespread. 

Anil et al. performed a network meta-analysis of intra-articular injections (steroids, ozone, saline, platelet-rich plasma, autologous conditioned serum, hyaluronic acid, botox, bone marrow aspirate concentrate, SVF, and MSCs) for OA knees with 79 RCTs consisting of 8761 patients. Of all the modalities analyzed, SVF had the highest P-score among the VAS (0.7676) and WOMAC (0.9044) scores at 12-month follow-up. At 1-year follow-up, they observed that intra-articular SVF injection demonstrated the highest potential in terms of pain relief and functional outcomes in OA knee patients [7]. 

Koh et al. performed second-look arthroscopy for patients who underwent SVF (4.04 × 10^6^ stem cells) for OA knees: 87.5% of patients showed the maintenance of suitable cartilage thickness with improved functional outcomes for at least 2 years [8]. The healing property of AD-derived SVF enhances cartilage regeneration, with attenuation of the pro-inflammatory cytokines and halting of OA progression. 

The systematic review and meta-analysis by Bolia et al. on the clinical efficacy of BMAC vs. SVF in OA knees includes 10 studies and 472 patients. Out of 472 patients, 233 patients received BMAC and 239 patients received SVF. The SVF group reported greater effects in terms of pain relief than the BMAC group, with a *p* value < 0.0001. An equivocal response was observed based on the functional outcomes of both groups. This study is limited by a publication bias in the available literature in terms of the isolation and characterization protocols. The SVF group (67%) outnumbered the BMAC group (50%) in terms of complications. The authors acknowledged the variability in cellular fraction isolation protocols; nevertheless, the greatest degree of pain relief was observed following a single intra-articular injection of SVF in long-term follow-up [9].

Shanmugasundaram et al. performed a systematic review of 11 studies and 4008 intra-articular SVF injections of knee joints. The method of SVF preparation varies, including ultrasonic cavitation, the use of collagenase 1 enzyme alone or with proteases, and combinations. The mean SVF cell count ranged from 0.39 × 10^6^ to 7.6 × 10^7^ with a viability of 78 to 91%. Most patients reported excellent pain relief and improved 6 m walking distance timings and functional outcome scores. None of the included studies reported donor-site morbidity or tumor formation, but one patient developed a knee joint infection following the SVF injection [10]. 

The systematic review of SVF injections of OA knees by Boada-Pladellorens et al. included nine studies published up to May 2021. All the studies reported pain relief and improved functional outcomes with WOMAC scores, but only five studies demonstrated changes on MR. All the studies used different protocols to isolate SVF from the adipose tissue, but the number of cells in the SVF mixture in each study varied greatly, introducing a comparison bias. However, in general, SVF was a safe, minimally invasive modality that could be used to manage knee OA patients [11].

These studies have several limitations:

(a) A lack of quantitative evidence available to assess cartilage healing via both MRI and second look arthroscopy. Additionally, only a few studies have undertaken biopsies to evaluate whether hyaline or hyaline-like cartilage was present.
(b) No control group to compare the efficacy of SVF injections against other available injectables.
(c) A lack of standardization in isolation, characterization, dosage, injection, and follow-up protocols for intra-articular SVF transplantation in OA knees.
(d) A lack of consensus on the autologous or allogenic source of SVF.

In conclusion, a few level 1 evidence studies are available regarding the use of SVF in knee OA patients. However, we should establish standardized protocols to prepare SVF according to the national regulatory requirements. In any case, on the proviso of the biases highlighted above, SVF seems to be a safe, minimally invasive modality for the management of knee OA patients. 
